# Sexual and reproductive health services for women with disability: a qualitative study with service providers in the Philippines

**DOI:** 10.1186/s12905-015-0244-8

**Published:** 2015-10-15

**Authors:** Kira Lee, Alexandra Devine, Ma. Jesusa Marco, Jerome Zayas, Liz Gill-Atkinson, Cathy Vaughan

**Affiliations:** Nossal Institute for Global Health, Melbourne School of Population and Global Health, The University of Melbourne, Victoria, 3010 Australia; Social Development Research Centre, De La Salle University, 2401 Taft Avenue, 1004 Manila, Philippines; Centre for Health Equity, Melbourne School of Population and Global Health, The University of Melbourne, Victoria, 3010 Australia

**Keywords:** Disability, Sexual and reproductive health, Violence, Women with disability, Accessibility, Disability discrimination, Philippines

## Abstract

**Background:**

The Philippines has ratified the United Nations Convention on the Rights of Persons with Disabilities and recently passed domestic legislation protecting the sexual and reproductive rights of people with disability. However women in the Philippines continue to report barriers to sexual and reproductive health services, and there is limited empirical evidence available to inform policy makers’ efforts to respond. This study aims to contribute to the available evidence by examining service providers’ perceptions of disability and their experiences providing sexual and reproductive health services to women with disability.

**Methods:**

The study was conducted as part of a larger three-year program of participatory action research that aims to improve the sexual and reproductive health of women with disabilities in the Philippines. Fourteen in-depth interviews and two focus group discussions were conducted with a total of thirty-two sexual and reproductive health service providers in Quezon City and Ligao. Qualitative data were analysed to identify key themes in participants’ discussion of service provision to women with disability.

**Results:**

Analysis of service providers’ accounts suggests a range of factors undermine provision of high quality sexual and reproductive health services to women with disability. Service providers often have limited awareness of the sexual and reproductive health needs of women with disability and inadequate understanding of their rights. Service providers have had very little training in relation to disability, and limited access to the resources that would enable them to provide a disability inclusive service. Some service providers hold prejudiced attitudes towards women with disability seeking sexual and reproductive health services, resulting in disability-based discrimination. Service providers are also often unaware of specific factors undermining the health of women with disability, such as violence and abuse.

**Conclusion:**

Recent legislative change in the Philippines opens a window of opportunity to strengthen sexual and reproductive health service provision across the country. However the development of services that are disability-inclusive will require substantial efforts to address supply-side barriers such as prejudiced service provider attitudes and limited capacity. Disability inclusion must be prioritised for the national goal of responsible parenthood and reproductive health to be realised for all.

## Background

An estimated 15 % of the world’s population lives with a disability, many of whom are disproportionately affected by poverty [1, 2]. People with disability face all forms of discrimination and exclusion from the social, cultural, political and economic life of their communities. Women with disability are acknowledged as experiencing unique and additional disadvantage because of intersectional discrimination associated with their gender and disability, resulting in a higher likelihood of experiencing exclusion compared with men with disability or women without disability [3–5]. This exclusion compromises a number of life outcomes for women with disability including education, employment, and attainment of health, including sexual and reproductive health (SRH).

## The Global context

The United Nations Convention on the Rights of Persons with Disabilities (CRPD) has specific provisions that recognise the reproductive rights of persons with disability (Art. 23); the right of people with disability to access SRH information and services (Art. 25); and the specific need for empowerment of women with disability (Art. 6) [6]. In order for these rights to be achieved, women with disability need to be provided with age appropriate, accessible information on SRH, and to have recognition of their rights to have a sexual relationship, marry, establish a family, enjoy reproductive health, and physical integrity [7, 8].

In low and middle-income countries, efforts to uphold these (and other) articles of the CRPD are hampered by a lack of data on the number of people living with disability and on their SRH needs and experiences [9, 10]. Evidence suggests women with disability have reduced access to health information, and experience barriers in accessing screening, prevention and care services resulting in greater unmet health needs, in particular in relation to SRH [3, 11].

Factors undermining the SRH of women with disability are multifaceted. The restricted economic participation of women with disability reduces their ability to access health services, compounded by a lack of locally available services and costly, inaccessible transport [3, 8, 12]. Even when services are available, a need for greater understanding on how service providers and the broader community can support the SRH of women with disability is required. Inaccurate and negative stereotypes circulate within community and health-care settings, including that women with disability are asexual, do not get married or have children, and therefore do not require SRH services [5, 7, 13, 14]. In addition service providers lack appropriate equipment, accessible educational materials, and have not had training on providing SRH services for women with disability [3]. Despite this, international development programs promoting access to SRH may inadvertently exclude women with disability as these programs are often not designed with due consideration of disability and the particular needs of people with disability [3, 9, 15].

Evidence suggests women with disability are two to four times more likely to experience physical and sexual violence (including intimate partner violence, abuse by other family members, rape, forced sterilisation, and/or abortion) than women without disability [4, 5, 16, 17]. Women with cognitive, communicative or psycho-social impairments are thought to be at particular risk [18]. The vulnerability of women with disability to violence is compounded by economic dependency; social isolation; the perception that women with disability will not report violence; women’s difficulties in physically defending themselves; and their exclusion from violence prevention programs [12, 19–21]. In addition to being a grave violation of their rights, violence against women with disability also undermines their health and SRH in particular.

### The Philippines context

The Philippines Statistics Authority estimate that 3.1 % of the population over the age of five has a disability [22]. Just under half (49.1 %) of all people with disability are women. Despite ratification of the CRPD in 2008 and the existence of legal frameworks that uphold the rights of women with disability to SRH and protection from violence, such as the Magna Carta for Women and the Magna Carta for Persons with Disability, Filipina women with disability continue to experience high rates of human rights violations. This is particularly the case for women with disability from low and middle-income groups [23].

One in five women aged between 15 and 49 years in the Philippines has experienced physical violence [24]. While these data are not disaggregated by disability, studies suggest that the rate of violence against women with disability is much higher [25]. However, the lack of accurate data on the prevalence of violence against women with disability (and on the prevalence of disability among women) makes it difficult to prevent and respond to the violence experienced by women with disability. Evidence from the Philippines is limited, but research elsewhere has found the capacity of SRH service providers to recognise and respond to incidents of violence against women with disability is limited, and connections between disability and violence prevention services are weak, undermining an effective response [26].

The primary legal framework for SRH in the Philippines is the Responsible Parenthood and Reproductive Health Act of 2012, commonly referred to as the RH Law, which mandates universal access to family planning including contraception, education and maternal care. However divisive debate about the RH Law, which is opposed by some of the larger faith-based institutions, has resulted in barriers to implementation and resourcing across the various levels of government [27, 28]. This has affected the provision of SRH services by local government units (LGUs), and undermined access to SRH services for all women including women with disability. SRH service delivery by LGUs is hampered by local pressures and opposition to the RH Law; difficulties in procurement of supplies; and a lack of skilled providers with capacity to deliver quality services [28].

Information on access to SRH services for women with disability in the Philippines is scarce [25]. A 2012 report commissioned by the United Nations Population Fund (UNFPA) suggested that agencies and service providers had little awareness of the SRH experiences of women with disability, and limited capacity to respond to their needs. The report highlighted the urgent need for more research to inform disability inclusive SRH policy and programming in the country, including the need to promote and further develop the capacity of service providers to respond to the specific needs of women with disability [25]. This article aims to contribute to the evidence available to SRH policy-makers and programs in the Philippines and elsewhere, reporting findings of in-depth interviews and focus group discussions conducted with SRH service providers in the initial phase of a three-year participatory action research program in the Philippines.

## Methods

Data presented in this paper were collected as part of W-DARE (Women with Disability taking Action on REproductive and sexual health), a three-year action research project aimed at improving access to quality SRH information and services, including the prevention of violence, for women and girls with disability in the Philippines. Researchers from the School of Population and Global Health at the University of Melbourne and the Social Development Research Center (SDRC) at De La Salle University in Manila lead W-DARE. Project partners include local Disabled People’s Organisations PARE, WOWLEAP, the national non-profit women’s health service provider Likhaan Center for Women’s Health, and the Center for Women’s Studies at the University of the Philippines. W-DARE is being implemented in Quezon City in Metro Manila, and in Ligao City in Albay Province and has involved the collection and analysis of data about the SRH experiences of women with disability in the Philippines, and the design, delivery and evaluation of interventions to increase access to SRH programs for women with disability [29]. The purpose of the study described in this paper was to investigate the knowledge, attitudes and practices of service providers in relation to the SRH of women with disability and to increase understanding about their experiences of providing SRH services to women with disability.

### Data sources

Face-to-face in-depth interviews and focus group discussions were conducted with health service providers across the two research sites. The question guides that were used in interviews and focus group discussions were developed and trialled in a participatory workshop involving co-investigators and W-DARE partners, and were informed by the experiences of women with disability. SDRC co-investigators and a representative from Likhaan conducted fourteen face-to-face in-depth interviews. SDRC co-investigators also conducted two focus group discussions, with a total of eighteen service providers participating. The average duration of the interviews was sixty minutes, and the two focus group discussions both lasted approximately ninety minutes. In both the interviews and the focus groups participants were asked about the services that they offered and whether these were used by women with disability; their perceptions of disability and SRH; and their experiences of and perceptions about the provision of services to women with disability.

Participants were purposively recruited from facilities and organisations providing SRH services in the two research sites. Any service provider involved in the delivery of SRH services to the public in Quezon City and Ligao City was eligible to participate, with recruitment being facilitated by the City Health Offices and W-DARE partner networks. Participants were (four male and twenty-eight female) service providers working for government and non-government organisations, and included doctors, nurses, midwifes, barangay (community) health workers, health service managers, and representatives of professional associations. Nineteen participants were from densely populated Quezon City (QC) and thirteen participants were from the largely rural Ligao City (LC). Data were collected in English or Tagalog, according to participant preference. Recruitment of participants ceased when descriptive saturation was achieved.

### Data analysis

Interviews and focus group discussions were audio-recorded with participants’ permission and transcribed and where necessary translated from Tagalog to English. Documentation of interviews that had been translated included the Tagalog transcription and the English translation side-by-side to facilitate quality checking of the translation by SDRC co-investigators. Data analysis was thematic and data driven [30], and drew upon the expertise and insights of co-investigators and W-DARE partners. The principal investigator (CV) facilitated a three-day data analysis workshop with co-investigators and W-DARE partners including women with disability, SRH service providers and gender specialists. Participants read and re-read the transcripts, identifying the key themes raised in the interviews and focus group discussions. An initial coding framework was collaboratively developed by workshop participants and refined by the first author (KL) during in-depth data analysis following the workshop. The coding framework was used to categorise and allocate data for each theme and was used as a template for storing and managing data in NVivo version 10.1.2.

### Ethics approval

All participants provided written informed consent to their participation in the study. Ethics approval for the study was obtained from the University of Melbourne Human Research Ethics Committee (Ethics ID 1340735.1) and the De La Salle University Ethics Committee in August 2013.

## Results

### Perceptions of disability and service provision for women with disability

Service providers’ perceptions of disability can be a significant barrier to them gaining a comprehensive understanding of, and responding to, the needs of people with disability (commonly referred to as “PWD” in the Philippines). The negative perceptions of some service providers was evident in their use of words such as “deficient”, “broken” and “inadequate” to describe people with disability, who were contrasted with “normal” people without disability.

A number of service providers were clearly uncomfortable talking about disability and people with disability, and were unclear of what terms were considered appropriate. Service providers’ attempts to avoid offending or disempowering people with disability through inappropriate language revealed their uneasiness and confusion when talking about disability.

*“It’s very disempowering if you say limitation, impairment. That’s why we use the word mentally challenged, with special concerns. Because the language itself would make them more of a loser. Right? So maybe that’s one, the language. We have to study the language” (FGD participant, QC)*

The disquiet that service providers expressed in relation to what language might be seen to be correct, was replicated in their attitudes towards provision of health services for people with disability. Service providers struggled with what they perceived to be the contradictory positions of acknowledging that people with disability should be prioritised to receive health services, and not wanting to ‘*discriminate*’ between people with and without disability for fear that this would be received as patronising.

*“It’s not specific because we treat everyone as a regular client whether it’s women, young girls, young women or young people. So it’s not exclusive for PWD because they might think that they’re not like the others” (FGD participant, QC)*

A number of service providers, including those in senior management roles in government services, stated that while people with disability may be ‘prioritised’ (the Magna Carta for Persons with Disability requires ‘prioritisation’) they will not receive any additional consideration.

*“Here in the Philippines, when you talk about social services or services that are being rendered by the government for free, there’s no distinction if you’re a person with disability or not…Their view is very much inclusive…When it comes to service, if women will be going to a health centre, for example and one is in wheelchair and one stands, just probably they might give a little preference to the person in the wheelchair, but they will just prioritise you, they will serve you first but after that priority, it stops there and there are no other considerations for that” (Government representative, QC)*

### Understanding of the SRH needs and rights of women with disability

Some service providers experienced particular difficulty discussing the SRH needs of women with disability during the interviews. The conservative socio-political context in the Philippines may have influenced participants’ willingness to discuss SRH, and the limited public discussion of the SRH needs of women with disability in the Philippines.

*“Well, among Filipinos as a whole, we are not talking about disabilities yet. It’s very difficult because Filipinos don’t want to talk about sex and all those things about having children or having menstruation or things like that. It is usually a taboo among women in the Philippines as a whole right? …So all the more when it comes to disability” (NGO representative, QC)*

Service providers often linked the right to, and need for, SRH services with marital status. One participant suggested that as women with disability in her catchment were married they would not need family planning services; conversely another stated that women with disability did not have a need for SRH services as they did not marry.

*“In my experience, those with disability in my community do not have husbands. But if I encountered someone with a husband or a family, I would have given them advice” (Midwife, LC)*

In addition to marital status, experiences of abuse were also linked to the right of women with disability to access SRH services. For example, one participant reported that women with disability, especially women with intellectual disability, did not have a need for family planning unless they experienced abuse.

*“Yes, they need medical health service but regarding family planning services, they wouldn’t really get married except if they are abused and get pregnant” (Nurse, QC)*

Some service providers reported that families of women with disability had requested tubal ligation for their daughter to prevent pregnancy. Service providers recognised that sterilisation of women with disability without their consent was a violation of women’s rights, however felt the practice was still sometimes justified in order to ease the burden on families and the strain on social services.

*“So those are the young women I handle, abused and with impairment. Especially if the impairment is cognitive, it’s very hard. Every time she comes to you, she’s pregnant. And it came to a point that the family doesn’t want to accept the child. And what they suggested is to ligate this woman because her first two children are already with the family and this is the third which will be given to them. And they cannot afford to have another baby. But we said, we don’t have the right to do that. But the relatives cannot understand it… But because of her inability to decide for her welfare, it was her mother and her relatives that decided for her… So you can see that we also have some ethical issues*” *(FGD Participant, QC)*

During the group discussions, only one respondent acknowledged women with disability are equally likely to have sexual desire and experiences as women without disability, and therefore require SRH services as standard practice.

*“But sometimes, they’re not innocent about sex already so it becomes their need. Even though they have disability, they’re still human” (FGD Participant, QC)*

While service providers did not report denying access to services for women with disability, their poor understanding of the SRH needs of women with disability undermines both their ability to provide services and the health-seeking behaviour of women with disability. One participant described the ignorance or prejudice (described as *“close-mindedness”)* of service providers, and of women with disability, as contributing to a lack of appropriate information being transferred.

*“Those whom make it hard are those close-minded service providers and also the close-minded clients. I feel that’s a major problem” (Doctor, QC)*

However there were service providers interviewed who recognised that women with disability needed SRH services and acknowledged the importance of assisting women with disabilities and their partners make informed choices regarding their SRH.

*“Personally, it seems they have need for such [SRH] services. They already have a disability, right? Chances are they may not be able to handle such things. So if they need such services and they want these services, it ultimately boils down to their choice, right? They will still be given a choice. We only inform them …if they would like to ask what information is available, and they are interested, they are the ones who will decide. Our role is to enable them to make an informed choice” (Nurse, LC)*

### Understanding of violence against women with disability

A number of the service providers interviewed were unaware that women with disability are more likely to experience violence than women without disability.

*“No, we have not had such a case that the raped woman had a disability or was beaten by the husband. They are not prone to that, those with disability” (Doctor, QC)*

Those service providers that did recognise the risk of violence experienced by women with disability had often had first hand experience of providing services in response to violence against a woman with disability. Service providers highlighted a number of factors as contributing to the vulnerability of women with disability to violence including homelessness, a lack of resources to meet basic needs, dependence on family members for care, and women being taken advantage of when family members were not present.

*“There is one case; sidecar drivers are taking advantage of the girl [with disability]; bringing her anywhere. They say that sometimes she sleeps in the public market” (Nurse, LC)*

Several service providers raised concerns about women with disability being sexually abused when family members (particularly mothers) were away from the home for work. However the concern often related to family or service provider anxiety about avoiding pregnancy as a result of abuse, rather than the abuse itself. Service providers recognised, and were concerned about, the additional stress families may experience if their family member with a disability became pregnant. However the right of women with disability to live safely in their own homes was rarely directly raised.

*“There was one family who had a mentally retarded child so they came in to ask what they should do with their child who has no supervision the whole day because they are working. They say doc, how’s this? Someone might take advantage of the child or violate her, what if she gets pregnant? Can she be put on family planning?” (Doctor, QC)*

A number of participants highlighted that women and girls with disability were particularly at risk of sexual abuse by male relatives within the home. However in discussing the abuse, service providers often framed disability as “the problem”, rather than the perpetrator’s criminal behaviour.

*“Sometimes there is incest … sometimes it is the brother who gets the sister pregnant; if not the brother, the father …. they take advantage of the weakness of the woman … it normally happens to them – in many cases, disability is the problem. Often, there are more of those with disability than those who are normal without disability” (FGD Participant, LC)*

Strategies to address the abuse of women and girls with disability that were suggested by service providers focused on prevention of pregnancy, rather than prevention of abuse. Prevention of both pregnancy and abuse was often seen as a mother’s responsibility, with one service provider suggesting:

*“Counselling to the mothers that they shouldn’t neglect [their children], that their child shouldn’t be out of their view. All throughout the day. Especially when she’s of menstruating age, she shouldn’t be out of your view because anytime of the day, anytime of the night, some dark-minded person might go in” (NGO Representative, QC)*

Other participants recognised that in addition to the family, the community and the State should have a role in protecting women with disability from violence, and in ensuring that women who had been abused had access to justice. Some service providers expressed frustration over the difficulties women with disability had in navigating the justice system.

*“What happens is it’s difficult to talk about it [sexual violence] because you’re Deaf. Like with our experience with NBI [National Bureau of Investigation], we brought a Deaf who is a rape victim and she can’t speak well. So there’s an interpreter. The NBI said, “Ah, no. It’s just a hearsay because that’s from a third person’s perspective, not the Deaf itself.” How can the Deaf speak? She’s Deaf, she can’t speak so it is her signs that is being interpreted by the interpreter to the NBI” (NGO representative, QC)*

One participant discussed a desire to institutionalise women with disability to “keep them safe”, indicating a lack of awareness of the high levels of abuse experienced by people with disability living in institutions globally.

*“I wish there would be an institution where women with mental impairment can be sheltered; for example, put in-house those who roam the streets; we have one here but it charges a monthly fee, which they cannot afford … They are abused by normal people, who have no mental impairment. The woman has a disability, then she walks outside; the men would rape her, then she gets pregnant” (FGD Participant, LC)*

On occasion, women with disability were reported as being responsible and at fault for negative SRH outcomes. Women with disability were blamed for unwanted or unplanned pregnancies, despite service providers perceiving women with disability to have low levels of SRH knowledge and awareness, and despite pregnancy often reported to be a result of rape.

*“Unfortunately, she got pregnant again. My role there as a social worker is that she came to me when she doesn’t have money to feed the baby. So she gave it to me. So what I did, I found foster family for the baby. After a year, she called me again, and she’s again pregnant. I did not entertain her anymore” (FGD Participant, QC)*

### Perceptions of barriers to SRH services for women with disability

Many participants perceived that the disadvantage of women with disability in the Philippines was not just because of their impairment, but was due to the intersection of discrimination based on gender, disability and poor socio-economic status. Service providers highlighted the conservative socio-political context in the Philippines as a factor undermining access to SRH for all women, including women with disability. Participants described how their ability to provide SRH services could be hampered by the socio-religious stance of the director or manager of the service in which they worked.

*“We do family planning advocacy. In the past we had a problem, because sometimes, it depends on the … head. But, we are okay this year. The previous years when we had a different head we weren’t, because [they were] pro-life” (Nurse, LC)*

Service providers also identified a range of factors specifically undermining the access of women with disability to SRH services and information, including the financial barriers that may be experienced by women with disability, and the limited financial, human and material resources available for service providers to provide appropriate services for women with disability. Service providers noted that there were often multiple structural barriers to services, combining to reduce access for women with disability.

*“First, the facilities are not accessible by the PWDs. Second, the front liners are not able to handle the needs of PWDs especially to the Deaf and to those with intellectual disabilities, and also the distance of health centre and health facility in the areas. The health centres are far and the transport is not accessible” (Government Representative, QC)*

*“In terms of the availability of supplies; because sometimes we lack supplies, and this becomes a problem. We have medical supplies, but there are times that they are depleted, they are not enough” (FGD Participant, LC)*

Participants also highlighted the need for more accurate data to inform the provision of SRH services to women with disability. Service providers felt that they did not have sufficient information about the SRH needs and experience of women with disability, and were unaware of how many women with disability lived in their local area or of how many women with disability actually used their services. Participants emphasised that it was difficult to compete with other government sectors for funding, or to advocate for more resources to increase disability inclusion in their service, without evidence to draw upon.

### Capacity of service providers

Many service providers felt that they lacked the capacity to provide appropriate SRH service to women with disability, stating that they had had no training in the area. Others felt that specialist care was required:

*“When they [health worker] see a PWD, they would say that they need a specialist to handle disability but there are none present especially in far flung rural areas. The health professionals there are midwife, nurse, only few doctors and they’re even general practitioners. And it’s also, there’s actually no, like for example, the health services to persons with disabilities, they have no training when it comes to rehabilitation services” (Government Representative, QC)*

Some participants noted that when service providers had limited training in how to meet the needs of women with disability, or held negative attitudes towards them, that clinicians could be uncomfortable treating women with disability and behave inappropriately. Descriptions of inappropriate behaviour included reports of verbal abuse.

*“As a service provider, instead of treating them [woman with disability] with a calm voice, it rises. They would usually shout at the patient. In the first place, he doesn’t know what are the things to ask. Instead of asking questions with reproductive intent, he might ask, “Why do you want to be pregnant?”” (FGD Participant, QC)*

Participants reported that communication difficulties were a specific barrier to service providers delivering SRH services to women with disability. Participants described the way that communication difficulties caused service providers to become frustrated, directly resulting in poor clinical management of women with disability.

*“I referred her [woman with psychosocial disability] to ER in a nearby hospital and she was not given a good treatment. She was shouted at because she’s not answering. See, [the doctor] didn’t give her a favourable treatment because she’s not answering. For me, because the patient is already bleeding, they should not have asked questions; they should’ve treated the complications when we rushed her there” (FGD Participant, QC)*

Service providers reported having particular difficulty understanding the needs of, and communicating information to, women who are Deaf or hard of hearing and women with intellectual disability. Trained sign language interpreters were rarely available to assist service providers, with service providers relying on family members.

“*I have difficulty explaining what I will be doing, like when I need to inject her for some medicine; that is our problem. We don’t know sign language; and it so happens that the mother doesn’t know sign language either; the only thing I know is that she’s pregnant, and she probably is aware that she needs to have a check-up; but we can’t understand each other. Because we have to get data, when her last menstruation was, neither the mother nor the daughter can provide the data; so what I did, I motioned through physical assessment to determine how many months is the baby in her womb; it’s really difficult” (FGD Participant, LC)*

Service providers also highlighted the additional time that may be required to communicate with women with disability, and noted that this was difficult in under-resourced workplaces.

*“You have to keep it simple for them but, of course, the discussion will take much longer. More time, more patients and more understanding [are needed] in dealing with PWD” (Nurse, QC)*

As in many other settings, factors undermining the capacity of service providers are anchored in broader weaknesses in health systems that require structural reform to address human resourcing requirements.

### The role of family in supporting women with disability access SRH services

A number of service providers discussed the key role that families play in either supporting or preventing access to SRH services, noting how the shame often associated with disability in the Philippines sometimes led to families “hiding” their family members with a disability, with obvious consequences for their health and well-being.

*“The obstacle has to do with the relative of the woman with disability. Because sometimes, they are hesitant or do not allow the woman with disability to go out of the house. They try to keep them inside the house, because they are ashamed if other people would know they have a relative who has a disability” (FGD Participant, LC)*

Service providers described families as, at times, acting as gatekeepers to SRH information and services, with the stigma associated with disability particularly undermining access to preventative programs.

*“Families of disabled even hide their own children, they do not bring them out…. Unless, something happens, then they come and look for help… They would come to us, to ask us what to do because their child was molested in school among their classmates, sometimes even their own teacher, it happens. So that’s when they ask questions” (NGO Representative, QC)*

Service providers also described how women with disability relied on family members to assist with transport to SRH services, and in covering service costs. For some women with disability from poor families, this financial dependence was a significant barrier to services.

*“Even if they have access, the relatives are burdened. They are not cooperative, they go home against medical advice because may be they don’t have the financials. And then if it will come to the end stage, they don’t have choice but to come back. And then sometimes they stay in the hospital for a very long time” (FGD Participant, QC)*

Family members often acted as intermediaries between women with disability and service providers, with service providers communicating with relatives rather than a woman with disability herself. In some instances this was because sign language interpreters were unavailable, or because service providers did not have sufficient skills to communicate with a woman with a communication or cognitive impairment. However, in other cases the rationale for communication through a relative was unclear.

*“If it’s a physical challenge, that’s no problem but if, let’s say, a mental challenge – you need to explain, be ready to understand, you need to explain to the parents. Another thing, it is difficult to communicate with the deaf and mute … When the blind come to the centre, they are usually accompanied so you can communicate directly with the companion*” *(Nurse, QC)*

Few participants reflected on how communication through a family member might inhibit a woman with disability seeking SRH services, or how this may render help-seeking for violence or abuse impossible if a family member was the perpetrator.

## Discussion

The perspectives and experiences of service providers described above reveal a range of barriers to SRH information and services for women with disability in the Philippines. Service providers highlight the difficulties women with disability may have in physically accessing facilities, in paying for health services, and in finding and paying for the accessible transport required to get to services. These barriers have been well-described in a range of settings [3, 15, 31], and addressing these physical and economic barriers underpins the Government of the Philippines’ Magna Carta for Persons with Disability. Findings from this study suggest there remains considerable work to be done to overcome these barriers to SRH for women with disability in the Philippines.

Disability discrimination by health service providers is known to undermine the health of people with disability globally [1] and addressing the discrimination faced by people with disability is another central element of the Magna Carta for Persons with Disability. However data presented in this paper highlight the discriminatory attitudes towards women with disability held by some service providers, and that these attitudes are often associated with limited understanding about disability and a lack of confidence in providing services for women with disability. Other service providers felt that women with disability should not receive ‘special’ treatment in order to avoid discriminating against them. This position was framed as non-discriminatory and inclusive, but does not recognise the additional barriers women with disability experience in accessing health services and that additional measures are therefore needed to increase equity of access.

Efforts to sensitise health service providers to the experiences of people with disability have been demonstrated to increase disability inclusion in health services elsewhere [3]. Our findings suggest that many SRH service providers in the Philippines would benefit from disability sensitisation to increase their awareness of factors undermining equity of access to services for women with disability. Incorporating disability sensitisation into the curriculum and initial training of service providers in the Philippines would greatly improve their capacity to appropriately include women with disability in mainstream SRH services. Similarly, awareness and sensitisation training would support current service providers to use appropriate language when discussing disability, challenge misconceptions about sexuality and disability, and ultimately strengthen their capacity to provide better quality SRH services for women with disability. Strengthened engagement with Disabled People’s Organisations would also increase service providers’ understanding of the day-to-day challenges facing women with disability when trying to access SRH information and services.

While some participants in this study were unaware that women with disability may experience violence, many service providers raised violence against women with disability and abuse by family members. International evidence confirms that violence against women with disability is common, with recent research conducted in the South East Asian and Pacific regions highlighting the impact of this on women’s health including their SRH [10, 16]. In discussing violence against women with disability, many service providers focused on the prevention of pregnancy arising from sexual abuse. The harms associated with unwanted and/or unplanned pregnancy are considerable, but short of holding parents (specifically, mothers) responsible, participants rarely discussed strategies to prevent the violence and abuse in the first place. This may reflect the professional orientation of SRH service providers, but the apparent lack of consideration of the right to safety for women with disability is concerning.

International agencies and human rights bodies have condemned the forced and coerced sterilisation of women with disability as a gross violation of human rights [32]. The practice has been documented around the world [13, 33–38] and was also raised by service providers in this study. Service providers recognised the challenges for women with disability and their families that arise from unwanted pregnancies, particularly in settings of poverty and over-stretched services. However alternative strategies to prevent unwanted pregnancy including accessible sexual health education, less permanent forms of contraception, or by preventing sexual coercion and abuse, were not raised in interviews. This suggests a particular priority for service provider education in the Philippines.

Service provider education and capacity development is also required to improve the ability of health workers to communicate effectively with women with disability. Participants described the negative impact of communication barriers on women’s SRH, in particular on the SRH of women who were Deaf or hard of hearing, or had cognitive or communication impairments. The number of sign language interpreters and resources for augmentative and alternative communication in the Philippines are completely inadequate to meet demand, so it is imperative that service providers learn at least basic skills to support their communication with women with disability. The suggestion made by some participants, that SRH services for women with disability should be provided by specialists, is impractical in a country where most of the population depend on services provided at local level by barangay (community) health workers, nurses, midwives and general practitioners. As with the wider population, women with disability need access to these services and have the right to receive quality local care.

Service providers correctly highlighted the lack of reliable disability prevalence data and research evidence as to the SRH needs and experiences of women with disability, including their experiences of violence. This study and the wider W-DARE program aim to contribute to the evidence base that policy makers and service providers can draw upon in the Philippines. Findings presented here suggest the need to ensure usage data from health and violence response services is disaggregated by disability to both increase understanding of women’s health needs and monitor disability inclusion of services. However it is also important that service providers and policy makers are not paralysed by the limited data available. Some participants in this study seemed to suggest that they were unable to consider strategies for disability inclusion in the absence of statistics. However the Philippines Magna Carta for Persons with Disability and the RH Law both contain very clear guidance on the responsibility of governments in relation to the provision of SRH and other services to women with disability.

The W-DARE project is using the findings from this study, and from research conducted with women with disability and their families, to inform the development and evaluation of pilot interventions to increase access to SRH services in two LGUs in the Philippines. Interventions include activities to build the capacity of SRH and violence response service providers, build the confidence and SRH knowledge of women with disability, support enabling local environments through LGU exchanges, and reduce the overall level of disability-based stigma and discrimination. Evaluation of these interventions will inform national efforts towards inclusive health systems.

### Limitations

The W-DARE project is focused on two specific LGUs in the Philippines, and the perceptions and experience of SRH service providers working in QC and LC may not reflect the views of service providers across such a diverse country. We report here findings from interviews and focus group discussions with SRH service providers but we did not engage with disability and rehabilitation specialists, or providers of institutional care to people with disability, who may also provide services to address their clients’ SRH needs. Analysis of data collected with women with disability and their families will be reported elsewhere.

## Conclusion

This study describes some of the significant challenges that service providers face in providing SRH services to women with disability in the Philippines. It also reveals service providers’ limited awareness in relation to disability, limited skills and capacity to deliver disability inclusive services, and in some cases discriminatory attitudes towards women with disability. The Philippines has ratified the UN Convention on the Rights of Persons with Disabilities and has passed legislation to protect the rights of people with disability including their right to SRH and protection from violence. Considerable effort and additional resources are required to enable Filipina women to realise their rights and for the aspirations of the Magna Carta for Disabled Persons and the Responsible Parenthood and Reproductive Health Law to be fulfilled.

## Abbreviations

CRPD: United Nations Convention on the Rights of Persons with Disabilities
LC: Ligao City
LGUs: Local Government Units
PWD: People with disability
QC: Quezon City
RH Law: Refers to the Philippines’ *Responsible Parenthood and Reproductive Health Act* of 2012
SDRC: Social Development Research Centre
SRH: Sexual and reproductive health
UNFPA: United Nations Population Fund
W-DARE: Women with Disability taking Action on REproductive and sexual health

## References

[1] WHO, World Bank (2011). World Report on Disability.

[2] Mitra S (2013). Data revolution for disability-inclusive development. Lancet Global Health.

[3] WHO, UNFPA (2009). Promoting sexual and reproductive health for persons with disabilities: WHO/UNFPA guidance note.

[4] Astbury J, Walji F (2014). The prevalence and psychological costs of household violence by family members against women with disabilities in Cambodia. J Interpers Violence.

[5] Stubbs T (2009). Pacific Sisters with disabilities: at the intersection of discrimination Fiji: UNDP Pacific Centre.

[6] United Nations. United Nations Convention on the Rights of Persons with Disabilities. http://www.un.org/disabilities/default.asp?navid=12&pid=150. 2006. Accessed Retrieved 15th June 2012.

[7] Schaaf M (2011). Negotiating sexuality in the convention on the rights of persons with disabilities. SUR International Journal on Human Rights.

[8] Foster K, Sandel M (2010). Abuse of Women with Disabilities: Toward an Empowerment Perspective.

[9] Morrison J, Basnet M, Budhathoki B, Adhikari D, Tumbahangphe K, Manandhar D (2014). Disabled women׳ s maternal and newborn health care in rural Nepal: a qualitative study. Midwifery.

[10] Astbury J, Walji F (2013). Triple Jeopardy: Gender-based violence and human rights violations experienced by women with disabilities in Cambodia.

[11] MacLachlan M, Swartz L (2009). Disability and international development: towards inclusive global health.

[12] Higgins D (2010). Sexuality, human rights and safety for people with disabilities: the challenge of intersecting identities. Sexual & Relationship Therapy.

[13] Frohmader C, Ortoleva S. The Sexual and Reproductive Rights of Women and Girls with Disabilities. ICPD International Conference on Population and Development Beyond; 2014.

[14] Mona LR, Cameron RP, Goldwaser G, Miller AR, Syme ML, Fraley SS (2009). Prescription for pleasure: exploring sex-positive approaches in women with spinal cord injury. Topics in Spinal Cord Injury Rehabilitation.

[15] Mall S, Swartz L (2012). Sexuality, disability and human rights: strengthening healthcare for disabled people. South African Medical Journal.

[16] Spratt JM. A Deeper Silence: The Unheard Experiences of Women with Disabilities and Their Sexual and Reproductive Health Experiences : Kiribati, the Solomon Islands and Tonga Fiji: UNFPA. 2013.

[17] Takamine Y. Disability Issues in East Asia: Review and Ways Forward2004 Contract No.: 2004–1.

[18] Jin-Ding L, Lan-Ping L, Pei-Ying L, Jia-Lin W, Chien-De L, Fang-Yu K (2010). Domestic violence against people with disabilities: prevalence and trend analyses. Research in Developmental Disabilities..

[19] Hughes K, Bellis MA, Jones L, Wood S, Bates G, Eckley L (2012). Prevalence and risk of violence against adults with disabilities: a systematic review and meta-analysis of observational studies. The Lancet.

[20] Shettle A. Women with disabilities in development: Intersecting invisibility, intersecting realities GPDD, World Bank. 2009.

[21] Ballan MS, Freyer MB (2012). Self-defense among women with disabilities: an unexplored domain in domestic violence cases. Violence Against Women.

[22] Philippines Statistics Authority. Disability prevalence confirmed by personal communication with Philippines Statistics Authority drawing on data in their Philippines in Figures 2014 national report. Personal communication with A. Devine. 2015.

[23] KAMPI, DPRI (2009). Monitoring the human rights of persons with disabilities in the Philippines.

[24] Philippines National Statistics Office. 2010 Census of Housing and Population. 2012.

[25] PARE. Qualitative Study on the Realization of Reproductive Rights and Protection from Violence for Women and Girls with Disabilities in the Philippines. Manila: UNFPA. 2012.

[26] Lund EM (2011). Community-based services and interventions for adults with disabilities who have experienced interpersonal violence: a review of the literature. Trauma, Violence, and Abuse..

[27] Centre for Reproductive Rights. Philippine Supreme Court Upholds Historic Reproductive Health Law., http://reproductiverights.org/en/press-room/Philippine-Supreme-Court-Upholds-Historic-Reproductive-Health-Law. 2014. Accessed 11th December 2014.

[28] Likhaan Center for Women’s Health (2014). Asian Pacific Resource and Research Centre for Women (ARROW).

[29] Vaughan C, Marella M, Edmonds T, Garcia J, Bisda K, Salgado J, et al. Doing action research: Perspectives on rationale, rhetoric, reality and results. Development Bulletin. 2014. (76 August 2014):6.

[30] Braun V, Clarke V (2006). Using thematic analysis in psychology. Qualitative research in psychology.

[31] Maxwell J, Belser J, David D (2007). A Health Handbook for Women With Disabilitites.

[32] WHO. Eliminating forced, coercive and otherwise involuntary sterilization: an interagency statement, OHCHR, UN Women, UNAIDS, UNDP, UNFPA, UNICEF and WHO. http://www.unaids.org/sites/default/files/media_asset/201405_sterilization_en.pdf: WHO2014.

[33] Dorozynski A (2000). Sterilisation of 14 mentally handicapped women challenged. BMJ.

[34] Mallet J, Kalambi V (2008). Coerced and forced sterilization of HIV-positive women in Namibia. HIV/AIDS Policy & Law Review / Canadian HIV/AIDS Legal Network.

[35] Yau MK, Ng GS, Lau DY, Chan KS, Chan JS (2009). Exploring sexuality and sexual concerns of adult persons with intellectual disability in a cultural context. British Journal of Developmental Disabilities.

[36] Nair P (2010). Recent develpoment: litigating against the forced sterilization of HIV-positive women: recent developments in Chile and Namibia. Harvard Human Rights Journal..

[37] Hamilton C (2011). Sterilisation, the UNCRPD and intellectually disabled girls and women: a suitable topic for discussion. Intellectual Disability Australasia.

[38] Zampas C, Lamackova A (2011). Forced and coerced sterilization of women in Europe. Int J Gynecol Obstet.

